# A review of the biomedical innovations for healthy longevity

**DOI:** 10.18632/aging.101163

**Published:** 2017-01-29

**Authors:** Alexey Moskalev, Vladimir Anisimov, Aleksander Aliper, Artem Artemov, Khusru Asadullah, Daniel Belsky, Ancha Baranova, Aubrey de Grey, Vishwa Deep Dixit, Edouard Debonneuil, Eugenia Dobrovolskaya, Peter Fedichev, Alexander Fedintsev, Vadim Fraifeld, Claudio Franceschi, Rosie Freer, Tamas Fülöp, Jerome Feige, David Gems, Vadim Gladyshev, Vera Gorbunova, Irina Irincheeva, Sibylle Jager, S. Michal Jazwinski, Matt Kaeberlein, Brian Kennedy, Daria Khaltourina, Igor Kovalchuk, Olga Kovalchuk, Sergey Kozin, Alexander Kulminski, Ekaterina Lashmanova, Ksenia Lezhnina, Guang Hui Liu, Valter Longo, Polina Mamoshina, Alexander Maslov, Joao Pedro de Magalhaes, James Mitchell, Arnold Mitnitski, Yuri Nikolsky, Ivan Ozerov, Elena Pasyukova, Darya Peregudova, Vasily Popov, Ekaterina Proshkina, Evgeny Putin, Evgeny Rogaev, Blanka Rogina, Jane Schastnaya, Andrey Seluanov, Mikhail Shaposhnikov, Andreas Simm, Vladimir Skulachev, Maxim Skulachev, Ilya Solovev, Stephen Spindler, Natalia Stefanova, Yousin Suh, Andrew Swick, John Tower, Andrei V. Gudkov, Jan Vijg, Andrey Voronkov, Michael West, Wolfgang Wagner, Anatoliy Yashin, Nadezhda Zemskaya, Zhaxybay Zhumadilov, Alex Zhavoronkov

**Affiliations:** ^1^ Moscow Institute of Physics and Technology, Dolgoprudny, 141700, Russia; ^2^ Department of Carcinogenesis and Oncogerontology, N.N. Petrov Research Institute of Oncology, St. Petersburg, Russia; ^3^ Pharmaceutical Artificial Intelligence Department, Insilico Medicine, ETC, B301, Johns Hopkins University, Baltimore, Maryland 21218, USA; ^4^ Bayer Global Drug Discovery, Berlin, Germany; ^5^ Social Science Research Institute, Duke University, Durham, NC 27708, USA; ^6^ School of Systems Biology, George Mason University, VA 20110, USA; ^7^ SENS Research Foundation, 1 Beaconsfield Terrace, Cambridge, CB2 3EH, UK; ^8^ Department of Immunobiology, Yale School of Medicine, New Haven, CT 06520, USA; ^9^ ActuRx, 92330 Sceaux, France; ^10^ Institute of Biology of Komi Science Center of Ural Branch of Russian Academy of Sciences, Syktyvkar, 167982, Russia; ^11^ Gero Limited, International Commerce Center, Kowloon, Hong Kong; ^12^ The Shraga Segal Department of Microbiology, Immunology and Genetics, Center for Multidisciplinary Research on Aging, Ben-Gurion University of the Negev, POB 653, 8410501, Beer-Sheva, Israel; ^13^ Department of Experimental, Diagnostic and Specialty Medicine (DIMES), University of Bologna, Bologna, Italy; ^14^ Department of Chemistry, University of Cambridge, Cambridge CB2 1EW, UK; ^15^ Department of Medicine, Research Center on Aging, Graduate Program in Immunology, Faculty of Medicine and Health Sciences, University of Sherbrooke, Sherbrooke, Quebec, Canada; ^16^ Nestlé Institute of Health Sciences, EPFL Innovation Park, 1015 Lausanne, Switzerland; ^17^ Institute of Healthy Ageing, and Department of Genetics, Evolution and Environment, University College London, London, UK; ^18^ Division of Genetics, Department of Medicine, Brigham and Women's Hospital, Harvard Medical School, Boston, MA 02115, USA; ^19^ Department of Biology, University of Rochester, Rochester, NY 14627, USA; ^20^ Nutrition and Metabolic Health Group, Nestlé Institute of Health Sciences, CH-1015 Lausanne, Switzerland; ^21^ Open Research Department, L'Oreal, 93600 Aulnay-sous-Bois, France; ^22^ Tulane Center for Aging and Department of Medicine, Tulane University Health Sciences Center, SL-12, New Orleans, LA 70112, USA; ^23^ Department of Pathology, University of Washington, Seattle, WA 98195, USA; ^24^ Buck Institute for Research on Aging, Novato, CA 94945, USA; ^25^ Department of the Integrated Prevention Programs, Federal State Institution “National Research Center for Preventive Medicine” of the Ministry of Healthcare of the Russian Federation, 101990, Moscow, Russia; ^26^ Department of Biological Sciences, University of Lethbridge, Lethbridge, Alberta, T1K 3M4, Canada; ^27^ Engelhardt Institute of Molecular Biology, Russian Academy of Sciences, Moscow, Russia; ^28^ National Laboratory of Biomacromolecules, CAS Center for Excellence in Biomacromolecules, Institute of Biophysics, Chinese Academy of Sciences, Beijing 100101, China; ^29^ Longevity Institute and Davis School of Gerontology, University of Southern California, Los Angeles, CA 90089, USA; ^30^ Department of Genetics, Albert Einstein College of Medicine, Bronx, NY 10461, USA; ^31^ Integrative Genomics of Ageing Group, Institute of Ageing and Chronic Disease, University of Liverpool, Liverpool, UK; ^32^ Department of Genetics and Complex Diseases, Harvard School of Public Health, Boston, MA 02115, USA; ^33^ Department of Medicine, Dalhousie University, Centre for Health Care of the Elderly-Suite 1305, 5955 Veterans' Memorial Lane Halifax, Nova Scotia, B3H 2E1 Canada; ^34^ Biomedical Cluster, Skolkovo Foundation, Skolkovo, Russia; ^35^ Institute of Molecular Genetics of Russian Academy of Sciences, Moscow, Russia; ^36^ Voronezh State University, Voronezh, Russia; ^37^ Computer Technologies Lab, ITMO University, St. Petersburg 197101, Russia; ^38^ Vavilov Institute of General Genetics RAS, Moscow, Russia; ^39^ Institute for Systems Genomics, School of Medicine, University of Connecticut Health, Farmington, CT 06030, USA; ^40^ Centre of Medical Basic Research, Medical Faculty, Martin Luther University Halle-Wittenberg, Halle (Saale), Germany; ^41^ Department of Bioengineering and Bioinformatics, Lomonosov Moscow State University, Moscow, Russia; ^42^ Department of Biochemistry, University of California at Riverside, Riverside, CA 92521, USA; ^43^ Institute of Cytology and Genetics of Siberian Branch of Russian Academy of Sciences, Novosibirsk, Russia; ^44^ Life Extension, Ft. Lauderdale, FL 33308, USA; ^45^ Department of Biological Sciences, University of Southern California, Los Angeles, CA 90089, USA; ^46^ Department of Cell Stress Biology, Roswell Park Cancer Institute, Buffalo, NY 14263, USA; ^47^ BioTime, Inc., Alameda, CA 94501, USA; ^48^ Helmholtz-Institute for Biomedical Engineering, Stem Cell Biology and Cellular Engineering, RWTH Aachen University, Aachen, Germany; ^49^ Center for Life Sciences Nazarbayev University, Astana, Kazakhstan

**Keywords:** longevity, aging, biomarkers, geroprotectors, epigenetics, transcriptomics

The field of biogerontology and regenerative medicine is rapidly evolving with many new advances in aging research promising to transform healthcare and extend healthy productive longevity. Recent breakthroughs in epigenetic, transcriptomic and multimodal biomarkers of aging, discovery of new and validation of old geroprotectors and advances in gene therapy provide an optimistic outlook. However, the propagation of laboratory advances into clinical practice has been comparatively slow with few focused investment and technology integration programs worldwide.

To evaluate the technological readiness of the biomedical advances in biogerontology and accelerate the translation from laboratory to clinical and commercial setting, a community of scientists with support from the innovative investment companies organized the third bi-annual international conference titled “Biomedical Innovation for healthy longevity” in Saint-Petersburg, Russia in 25-27 of April 2016. The conference was organized by **Alexey Moskalev**, A**lex Zhavoronkov**, **Vladimir Anisimov**, **Olga Tkacheva** with support from the Fomenko family including **Andrei Fomenko**, **Lada Fomenko** and **Isabel Fomenko** acknowledged in this paper and attended by over 400 scientists from over 20 countries, representing the largest biomedical centers and presenting their work.

The conference included two parallel tracks covering research and translational aspects of aging research and three open round tables on longevity journalism, new methods of financing aging research and classifying ageing as a disease in the context of International Classification of Diseases 11th Revision (ICD-11) chaired by **Alex Zhavoronkov**, **Alexey Moskalev** and **Daria Khaltourina** with over 30 prominent scientists and medical doctors present. While most roundtable participants agreed that it is important to classify ageing as a disease to attract attention and resources to the field and make preventative treatments reimbursable with specific codes, there was major disagreements on the research, medical, ethical, regulatory and business issues. Two main approaches for classifying ageing as a disease were considered: assigning ageing a separate actionable code and developing a taxonomy of age-associated conditions with separate codes. The roundtable concluded with the proposal to develop an open community-managed discussion group using a common MediaWiki engine at http://www.ICD-11.org to explore the various approaches, aggregate the proposals and engage in international collaboration on both the ICD-11 and other disease classification registries.

Over 60 scientists gave talks, presented posters and submitted abstracts for the conference.

**Brian Kennedy** (Buck Institute for Research on Aging, USA) presented “Gender-specific effects of the mTOR pathway on metabolism and aging”. **Claudio Franceschi** (University of Bologna) reported “Decelerated aging in semi-supercentenarians and their offspring according to the epigenetic clock”. **Andrei V. Gudkov** (Roswell Park Cancer Institute) presented “Origin, biological significance and pharmacological targeting of senescent cells in vivo”. **Vishwa Deep Dixit** (Yale School of Medicine) presented “Inflammasome and age-related inflammation”.

**Igor Kovalchuk** (University of Lethbridge) in his report “Epigenetics of aging – from mechanisms to preventative strategies” presented an epigenetic theory of aging and the novel modes of epigenetic anti-aging interventions. Cells in the aging body exhibit extensive differences to cells in the juvenile organism, ranging from changes in gene expression and DNA methylation patterns, shortened telomeres, to the deteriorating genome maintenance mechanisms. At the tissue, organ and systemic level, cells are exposed to the altered stromal milieu caused by the altered secretory profiles of senescent cells and the deteriorating immune system which is not equally able to mount an immune response towards new antigens. The combination of these factors may facilitate the malignant transformation of cells and prevent the efficient recognition and clearance of transformed cells, thus leading to cancer predisposition.

**Wolfgang Wagner** (Helmholtz-Institute for Biomedical Engineering) presented “Simple Epigenetic Biomarkers for Biological Age”. Aging is reflected by specific modifications in the DNA-methylation (DNAm) pattern. These epigenetic changes are enriched in developmental genes and can be reversed by reprogramming into induced pluripotent stem cells (iPSCs). Apparently, iPSC-derived cells remain epigenetically rejuvenated. Age-associated hyper-methylation seems to be coherently modified in cancer and may reflect chromosomal reorganization that favors tumor-initiating genomic mutations. We established a simple signature based on DNAm levels at only three specific CpG sites (associated with the genes *PDE4C*, *ASPA*, and *ITGA2B*). This approach facilitates age predictions of blood samples with a mean absolute deviation from chronological age (MAD) of less than five years. The method is also applicable for buccal swabs, which facilitate non-invasive harvesting of DNA. However, the models need to be adjusted for this different specimen and the precision of age-predictions can be increased by using an additional cell-type specific signature - a “buccal-cell-signature” - that reflect the composition of buccal epithelial cells and leucocytes. Notably, there is evidence that simple epigenetic signatures can also be indicative for of all-cause mortality in later life. These results hold the perspective that simple epigenetic biomarkers, based on few or individual age-associated CpGs, can assist estimation of biological age.

**Jan Vijg** (Albert Einstein College of Medicine) “Genome instability: A conserved mechanism of Aging?”. DNA mutations in somatic cells have since long been considered as a possible causal contributor to aging. However, since somatic mutations are rare they can only be detected in clonal lineages, such as tumors or organoids. We developed a reliable, validated assay to detect mutations across the genome in single cells or nuclei after whole genome amplification. Using this assay we discovered that mutation frequency in somatic cells is much higher than in the germline, confirming the disposable nature of the soma. Our results also show an age-related increase in somatic DNA mutations in human white blood cells. We plan to use the single-cell genomics assay to study the landscape of somatic mutations in different organs and tissues of aging humans.

**Yousin Suh (**Albert Einstein College of Medicine) reported “Functional genomics approach to develop targets for slowing aging in humans”. The discovery of evolutionarily conserved pathways with major impact on lifespan and health span in animal models has suggested a potential to identify therapeutic targets for interventions that could favorably influence age-related outcomes in humans. Identification of gene variants that protect humans against crippling diseases at old age is likely to help find novel strategies for prevention and therapy. With the advent of novel high-throughput genomic technologies, discovery of functional pathways that influence lifespan or health span in humans is now feasible through identification of genetic variants associated with these outcomes. Since longevity is associated with diminished risk for diseases and pathologies, novel potential therapeutic targets could be identified based on knowledge of functional pathways of gene variants associated with longevity. We have been conducting systematic multidisciplinary studies to discover functional gene variants associated with longevity in the conserved pathways of aging, including (epi)genome maintenance, using functional genomics approach. To understand molecular mechanisms underlying the association with longevity, functional consequences of gene variants on age-related parameters are assessed in cell culture and in vivo animal models. The results may open up the possibility of targeted and personalized intervention strategies, ultimately leading to improved quality of life of the elderly population.

**Guang-Hui Liu** (Institute of Biophysics of Chinese Academy of Sciences) presented “Using stem cell and gene editing techniques to study and treat human genetic disorders”. Gilford progeria syndrome (HGPS) and Werner syndrome (WS) are two human premature aging disorders with features that closely recapitulate the features of human ageing. Mutations in *LMNA* and *WRN* genes lead to aberrant splicing product progerin and protein loss in HGPS and WS, respectively. Study on how genetic alteration leads to the cellular and organismal phenotypes of premature aging will provide clues to the molecular mechanisms that underlie physiological ageing and increase our understanding of molecular pathways contributing to healthy aging. We have generated induced pluripotent stem cells (iPSCs) from fibroblasts obtained from patients with HGPS. Further, using targeted gene correction technology, we successfully corrected the mutated *LMNA* gene in HGPS-iPSCs. Finally, by using targeted “knock-out” and “knock-in” technique, we also created WS-specific human embryonic stem cells (hESCs) with *WRN* mutation as well as Parkinson's disease (PD)-specific hESCs with *LRRK2* mutation. Upon differentiation of these “diseased” human pluripotent stem cells into different somatic cell types, they demonstrated aging-associated tissue-specific disease phenotypes. Together, these tools offer unprecedented platforms to study the pathogenesis of human aging and aging-related diseases.

**Vadim Gladyshev** (Harvard Medical School) “Lifespan control across species and model systems”. Many human diseases are associated with aging, which is often their most significant risk factor. The aging process can be regulated during evolution, e.g. mammals show >100-fold difference in lifespan. We employ this diversity to shed light on mechanisms that regulate lifespan. For this, we apply comparative genomics to short- and long-lived species and carry out analyses across panels of mammals. We sequenced the genomes of several mammals with exceptional lifespan and identified genes that may contribute to their longevity. In addition, we carried out analyses of gene expression, metabolites and elements across large panels of mammals. We also analyzed gene expression across different cell types that are characterized by different longevity (cell turnover). These studies point to both unique (to cells, lineages) and common adaptations to longevity involving various pathways. It is our hope that a better understanding of molecular mechanisms of mammalian lifespan control will lead to a better understanding of human diseases of aging.

**Andrey Seluanov** (University of Rochester) “Longevity mechanisms in the naked mole rat and other long-lived mammals”.

**John Tower** (University of Southern California, Los Angeles) “Sex-specific regulation of life span in Drosophila”. Aging in Drosophila is associated with up-regulation of the innate immune response, the oxidative stress response, and the proteotoxicity response, including the mitochondrial unfolded protein response (UPRmt); these changes suggest an aging-associated failure in mitochondrial maintenance that limits life span. Accordingly, we found that transgenic reporters for genes of innate immune response (antimicrobial peptide/AMP genes), cytoplasmic UPR (Hsp70) and UPRmt (Hsp22) are predictive biomarkers of life span. Mifepristone/RU486 is a glucocorticoid receptor antagonist and progesterone receptor antagonist with human female contraceptive and abortifacient activities, reported to reduce inflammation. In female Drosophila, mating increases reproduction and inflammation and decreases life span. We found that mifepristone/RU486 acts in Drosophila females to decrease reproduction, delay inflammation and increase median life span up to +68%. Long-lived females had normal or increased food consumption based on dye-uptake and capillary-feeding assays, arguing against a dietary restriction mechanism. Both mating and mifepristone/RU486 changed median life span by altering initial mortality rate. High-throughput RNA sequencing was used to identify genes up-regulated or down-regulated upon mating, and where the change was reduced by mifepristone/RU486. Several candidate positive regulators of life span were identified that are conserved in humans, including dosage compensation regulator Unr and the Dopamine 2-like receptor. Candidate negative regulators included neuropeptide CNMamide and several involved in protein mobilization and immune response, including the AMP gene Drosocin. Analysis of Drosocin-GFP reporters in live flies recapitulated the aging-associated inflammation, including the effects of mating and mifepristone/RU486. The results implicate steroid hormone signaling in regulating sex-specific trade-offs between reproduction versus immune function and longevity.

**Vadim Fraifeld** (Ben-Gurion University of the Negev) “Mitochondria: a bottleneck of aging and longevity?” Nuclear-mitochondrial relationships could be characterized as “enslaving” rather than symbiosis. Indeed, mitochondria are the most “hard-working” organelles in the animal cell, which have delegated the vast majority of the genes to the nuclear genome. This situation inevitably brings about to a “conflict of interests”, with far-reaching consequences. Unsurprising-ly, mitochondria-associated variables (mtDNA GC content, metabolic rate, metabolic score, body temperature) are powerful predictors of mammalian longevity, and thus could be considered the main targets for longevity-promoting interventions.

**Vera Gorbunova** (University of Rochester) “The mechanisms of more efficient DNA repair in long-lived mammals: the role of SIRT6”.

**Blanka Rogina** (University of Connecticut Health) “*Indy* reduction maintains fly health and homeostasis”. *Indy* (*I'm not dead yet*) encodes the fly homologue of a mammalian transporter of the Krebs cycle intermediates. Reduced *Indy* gene activity has beneficial effects on energy balance in mice, worms and flies, and worm and fly longevity. In flies, longevity extension is not associated with negative effects on fertility, mobility or metabolic rate. Others and we show that *Indy* reduction extends longevity by mechanism similar to calorie restriction (CR). Some of the hallmarks of these changes are altered intermediate nutrient metabolism, increased spontaneous physical activity and increased mitochondrial biogenesis. These changes have been found in fly heads, thoraces and the midguts. The observed changes in midgut energy metabolism, specifically decreased production of free radicals, results in preservation of intestinal stem cell (ISC) homeostasis and midgut integrity. Our studies show a direct link between changes in energy metabolism, caused by the *Indy* reduction and preservation of ISC homeostasis. The data suggest that *Indy* reduction preserves homeostasis in tissues that contribute to extended health and longevity.

**Elena Pasyukova** (Institute of Molecular Genetics of Russian Academy of Sciences, Russia) “Neuronal transcriptional regulators of lifespan in Drosophila melanogaster”.

**Irina Irincheeva** (Nestlé Institute of Health Sciences) presented “Why don't we all lose weight equally on Caloric Restriction?” Proteomics explanation to weight loss variability on a low-calorie diet in overweight and obese subjects” Obesity is characterized by a state of metabolic inflexibility and chronic inflammation leading to the development of comorbidities like type 2 diabetes, dyslipidemia or certain cancers and generally to a decreased life expectancy in obese individuals. Low calorie diets (LCD) (<1000 kcal per day) have been shown to be very effective in improving many of the metabolic dysfunctions. However, the capacity to lose weight and the associated metabolic improvements show significant variability in humans, even under the same controlled dietary regimes. To understand the molecular basis for these differences we screened the plasma expressions of over twelve hundred proteins in 500 overweight and obese subjects to determine whether we could predict at baseline the weight loss outcome of 8 weeks LCD diet (800 kcal per day). As discovery data to construct a weight loss predictive model we used the Pan-European cohort DiOGenes (Diet, Obesity and Genes, Larsen et al., 2010). To select weight loss predictive proteins we deployed elastic net bootstrap estimation of high-dimensional regression (Chatterjee and Lahiri, 2011) adjusting for gender, age and BMI at baseline. We evaluated the accuracy of our predictive model on the data set of 500 independent subjects from Ottawa Hospital Weight Management Clinic using LCD for weight loss. The accuracy of the predictive model was significantly higher than random in the independent data set.

To identify functional relationships and biochemical pathways shared between the predictive proteins we performed network analyses. The results allow us to formulate a first hypothesis on biological processes leading to successful weight loss for overweight and obese subjects on a low-calorie diet.

**Alexander Kulminski** (Duke University) presented “Uncoupling associations of risk alleles with endophenotypes and phenotypes: Insights from the Apolipoprotein B locus, lipids, myocardial infarction, and survival”. Traditionally, genome-wide association studies (GWAS) have emphasized the benefits of large samples in the analyses of age-related traits rather than their specific properties. We adopted a realistic concept of genetic susceptibility to inherently heterogeneous, age-related traits driven by the elusive role of evolution in their properties. We analyzed in detail the associations of rs693 and rs562338 polymorphisms representing the Apolipoprotein B locus with endophenotypes (total cholesterol [TC] and high-density lipoprotein cholesterol) and phenotypes (myocardial infarction [MI] and survival) in four large-scale studies. We showed that a strong, robust predisposition of rs693 and rs562338 to TC (beta=0.72, p=7.7×10-30 for rs693 and beta=-1.08, p=9.8×10-42 for rs562338) is not translated into a predisposition to MI and survival. The rs693_A allele influences risks of MI and mortality for MI patients additively with lipids. This allele shows antagonistic effects -- protecting against MI risks (beta=-0.18, p=1.1×10-5) or increasing MI risks (beta=0.15, p=2.8×10-3) and mortality for MI patients, in different populations. Paradoxically, increased TC concentrations can be protective against MI for the rs693_A allele carriers. Our results uncouple the influences of the same alleles on endophenotypes and phenotypes despite potential causal relationships among the latter. Our strategy reveals an overall highly significant association of rs693 with MI (p=5.5×10-8) that is contrasted with a weak estimate following the traditional, sample-size-centered GWAS strategy (p=0.16) in the same sample. These results caution against the use of the traditional GWAS strategy for gaining profound insights into genetic predisposition to healthspan and lifespan.

**Vladimir Skulachev** (Moscow State University) in his lecture “Naked Mole Rats and Humans: Highly Social Creatures Prolonging Youth by Delay of Ontogenesis (Neoteny)” considered some physiological mechanisms responsible for longevity of eusocial mammals, i.e. a rodent (naked mole rat) and a primate (human). It is concluded that both naked mole rat and human are no more affected by dynamic natural selection due to specific organization of the socium (naked mole rat) and substitution of fast technical progress for slow biological evolution (human). Since aging is supposed to be a program stimulating evaluability by increasing pressure of natural selection upon an individual, such a program became a harmful atavism for naked mole rat and human. This is apparently why aging as a reason for death is very rare in naked mole rats younger than 30 years and humans younger than 55 years. Such an effect is achieved, at least partially, by prolongation of youth (neoteny). The numerous facts are described indicating that The Master Biological Clock responsible for timing of ontogenesis is retarded both in naked mole rat and in human. In these species, numerous traits of youth do not disappear (or disappear enormously slowly) with age. For a long time, in naked mole rat, this point of view was supported mainly by morphological observations, such as absence of hair, auricles and scrotum, underdevelopment of lungs, etc. Recently, numerous physiological features of neoteny in naked mole rat were described. Among them are (i) long gestation, (ii) long maturity time, (iii) strong delay in brain development, (iv) regeneration and elongation of neurons during entire life span, (v) extremely high resistance of adult mole rat neurons to anoxia/reoxigenation, a property inherent in newborn and young, but not adult mammals, (vi) resistance to H2O2 - induced apoptosis of cell culture, (vii) absence of age-linked decay in levels of antioxidants (in particular, due to very high concentration of extracellular antioxidant hyaluronan), (viii) absence of any increase in peroxidation index of lipids with age, (ix) no age-linked increase in ROS production, (x) retarded postnatal development of mitochondrial reticulum in skeletal muscles, (xi) absence of decay in the amount of mitochondria with age, (xii) no age-induced decay in proteasome level, (xiii) no indications of aging of immune system, (xiv) low transcription of genes and activity of insulin and IGF1 and high transcription and activity of IGF2, etc. These and other specific features of the naked mole rats explain their resistance to cancer, infections, cardiovascular and brain diseases, diabetes, and, as a result, their long maximal lifespan, which is more than 30 years vs. 3.5 years for mice (a rodent of the similar size).

For humans, it is generally accepted that embryo, neonatanal and young organisms have features similar to other primates, thereby the listed morphs resemble very much human, not ape. As to the ape-specific traits (a lot of hair on the body, construction of the skull, large superciliary arches, etc.), they appear in adulthood of animals and do not appear in humans. Recently, studies of brain transcriptomes of humans, chimpanzees and rhesus macaques revealed that in humans, just like naked mole rats, transcription of large group of genes is strongly retarded compared to the great apes. Comparison of pairs of highly and lowly social mammals (naked mole rat vs. mouse and human vs. chimpanzee) is very interesting. In both pairs, highly social representative (i) is long-lived, (ii) its age-dependent mortality during first 30-55 years is so low that its contribution to the total mortality is negligible, (iii) gestation and maturation times are longer, (iv) brain development is strongly retarded but without negative effect on the final mass of the organ, (v) skeletal muscle development is also retarded resulting in lowering in the final mass.

Demonstration of neoteny in humans at physiological and genetic levels is very important for understanding of physiological, pathological and therapeutic aspects of aging. In particular, prolongation of youth by delay of aging is impossible to imagine within the framework of the concept of stochastic (non-programmed) aging but can easily be explained if aging is programed and controlled by The Master Biological Clock, like other main steps of ontogenesis. Arrest of operation of aging program in humans by an antiaging medicine seems to be a promising approach to prolong our healthspan.

**David Gems** (University College London) spoke on “The origins of senescent pathology in *C*. *elegans*”. The biological mechanisms at the heart of the aging process are a long-standing mystery. An influential theory has it that aging is the result of an accumulation of molecular damage, caused in particular by reactive oxygen species (ROS) produced by mitochondria. This theory also predicts that processes that protect against oxidative damage (involving detoxification, repair and turnover) protect against aging and increase lifespan. However, recent tests of the oxidative damage theory, some using the short-lived nematode worm *C. elegans*, have often failed to support the theory. This motivates consideration of alternative models. One new theory, conceived by M.V. Blagosklonny and based on the antagonistic pleiotropy theory of G.C. Williams, proposes that aging is caused by the non-adaptive running on in later life of developmental and reproductive programmes. Such quasi-programmes (i.e. that are genetically programmed but non-adaptive) give rise to hyperfunction, i.e. functional excess due to late-life gene action, leading via dysplasia (including hypertrophy and hyperplasia, and atrophy) to the age-related pathologies that cause the late-life increase in mortality. Here we assess whether the hyperfunction theory is at all consistent with what is known about *C. elegans* aging, and conclude that it is. In particular, during aging *C. elegans* show a number of changes that may reflect pathology and/or hyperfunction, including oocyte hypertrophy to form tumor-like masses, proximal gonad atrophy and disintegration, massive yolk accumulation, cuticular hypertrophy and neurite outgrowth. Such changes are retarded in long-lived mutants, and can contribute to mortality. We demonstrate how futile run-on of yolk synthesis, conversion of intestinal biomass into yolk, and germline apoptosis generate late-life pathology. Our assessment implies that the hyperfunction theory is sufficient to explain the origins of major senescent pathologies in *C. elegans,* i.e. is a major cause of aging in this organism. The relative importance of hyperfunction and molecular damage as causes of different senescent pathologies in different organisms, and the interactions between these two major senescent etiologies are important topics for future investigation.

**Vadim Gladyshev** (Harvard Medical School) “The rising deleteriome”. Understanding the nature of aging is an important step in developing approaches to manipulate it. Various theories posit that aging may be caused by molecular damage, genetic programs, continued development, hyperfunction, antagonistic pleiotropy alleles, mutations, trade-offs, incomplete repair, etc. I will discuss that these ideas can be conceptually unified as they capture particular facets of aging. Living is associated with a myriad of deleterious processes, both random and deterministic, which exhibit cumulative properties, and represent the indirect effects of biological functions at all levels, from simple molecules to systems. From this, I derive the deleteriome, which encompasses cumulative deleterious age-related changes and represents the biological age. The organismal deleteriome consists of the deleteriomes of cells, organs, and systems, which change along roughly synchronized trajectories and may be assessed through biomarkers of aging. Aging is then a progressive decline in fitness due to the increasing deleteriome, adjusted by genetic, environmental, and stochastic processes. I will discuss how the deleteriome can be analyzed by following increased molecular damage in the form of low molecular weight species, somatic mutations derived from the analysis of cancer genomes, and dietary approaches.

**Arnold Mitnitski** (Dalhousie University) reported “A dynamical network model for age-related accumulation of health deficits and mortality”. How long people live depends on their health, and how it changes with age. Individual health can be tracked by the accumulation of age-related health deficits. The fraction of age-related deficits is a simple quantitative measure of human aging. This quantitative frailty index is as good as chronological age in predicting mortality. It has been shown that the accumulation of deficits is related to age associated imbalance between damage and repair processes, at the different levels of the organism. What causes such an imbalance was not clear. In order to answer this question, we developed a dynamical network model of aging. An individual was represented by a network of connected deficit nodes (n=800), each of which has two stable states corresponding to health and damaged. In our model, damage and repair rates have no explicit time-dependence, but do depend on the state of connected nodes. Transitions between the states were governed by: (i) interactions between the nodes, i.e. network connectivity, and; (ii) the environment, with a general stochastic mechanism (white noise) representing the environment. With this model, we observed upward curvature of the frailty index and broadening the distribution of the frailty index with age. We use a simple mortality criterion, where mortality occurs when the most connected node is damaged. In this way, we qualitatively reproduce Gompertz's law of exponential increasing human mortality with age. No explicit time-dependence in damage or repair rates was needed in our model. Instead, implicit time-dependence arose through deficit interactions - so that the average deficit damage rates increased, and deficit repair rates decreased, with age. We discuss the implication of our computational model that will allow us to start with high-quality model data, before we test our insights against current and emerging clinical data.

**S. Michal Jazwinski** (Tulane University Health Sciences Center) presented “Metabolic and Genetic Markers of Biological Age”. Biological and chronological age are not the same, as individuals depart in health from the average. Taking a systems approach, we developed an objective measure of healthy aging, a frailty index (FI34) composed of 34 health and function variables. FI34 is a much better predictor of mortality than is chronological age; therefore, it directly reflects biological age. It increases exponentially with chronological age, but it does so more slowly for offspring of long-lived parents. FI34 is also heritable (h2=0.39). Thus, it can be used in genetic analyses. The patterns of aging described by the variables in FI34 are very different for offspring of long-lived and short-lived parents. We have examined the association of the components of energy metabolism with FI34 in the oldest-old. Surprisingly, there is a positive association of FI34 with resting metabolic rate (RMR). This points to the rising cost of maintenance of integrated body function with declining health during aging. There are differences between males and females, however. In males, circulating creatine kinase (CK) increases with FI34. A decline in fat-free mass (FFM) is found instead in females. The CK increase in males is associated with variation in the genes XRCC6 and LASS1. These genes have in common a role in cell death, suggesting tissue damage as a source of increased energy costs of maintenance with declining health in males. In females, there is an association of variants of UCP2 and UCP3 with FI34, which is not found in males. These genes encode mitochondrial membrane transporters that impact cell metabolism. An interaction of a functional variant of UCP3 with RMR explains the association with FI34. We have used FI34 in an unbiased screen for genomic variation associated with healthy aging. This uncovered a region on chromosome 12 associated with this phenotype. Fine-mapping dissects this into three sites, which bear marks of regulatory function and association with longevity. Supported in part by grants from the National Institutes of Health (NIH).

**Aubrey de Grey** (SENS Research Foundation) spoke on “Longevity escape velocity: incorporating technological progress into extrapolation”. Predictions of future longevity have historically failed, by and large, to prove accurate. I will argue that this is because they are in one or another way based on pure extrapolation of past trends, a method that incorporates the implicit assumption that it does not matter *how* we contrived to achieve longevity improvements. I will explain how a careful examination of the medical and other advances that led to increased longevity could have resulted in much better predictions of their impact. I will then focus on the future, and especially on the initially counterintuitive but ultimately inescapable conclusion that regenerative medicine applied to the health problems of old age will at some point, probably within the next few decades, create a sharp discontinuity - which others have termed the “Methuselarity” - in the longevity of successive cohorts. This discontinuity will be so dramatic that period life expectancy will literally cease to be calculable, because mortality rates for all ages so far attained will become so low that survival probabilities will multiply to more than 50%.

**Daniel Belsky** (Duke University) presented “Quantification of biological aging in young adults”. Population aging threatens to bring a tidal wave of disease and disability (1). Strategies to prevent or treat individual diseases will be inadequate to contain costs and preserve economic productivity; interventions that address the root cause of multiple diseases simultaneously are needed. Such “geroprotective” interventions are emerging from model organism studies. Translation of these interventions to humans is slowed by the need for lengthy follow-up to evaluate effectiveness. This is especially true in the case of interventions that will need to be applied to young humans free of age-related disease, for whom prevention is still possible. Thus, surrogate endpoints for trials of geroprotective interventions are needed. Because most human aging research examines older adults, many with chronic disease, little is known about aging in young humans. We studied aging in 954 young humans, the Dunedin Study birth cohort. To quantify biological aging in these individuals, we tracked multiple biomarkers across three time points spanning their 20s and 30s. We devised a longitudinal measure that quantifies the pace of coordinated physiological deterioration across multiple organ systems (e.g., pulmonary, periodontal, cardiovascular, renal, hepatic, and immune function). This measure, the “Pace of Aging,” showed substantial variation in young, healthy adults who had not yet developed age-related disease. Young adults with faster Pace of Aging were, by midlife, less physically able, showed cognitive decline and brain aging, self- reported worse health, and looked older. The number of assays and repeated measurements required to assess Pace of Aging make it impractical for large-scale geroprotector trials. Biological aging measures that can be implemented in a single cross-section of data at baseline and follow-up are needed. We examined Pace of Aging alongside several, more scalable cross-sectional measures of biological aging. These measures were based on clinical biomarker and genomic data, including two recently published clinical-biomarker algorithms, telomere length, and epigenetic clocks. We asked two questions: (1) Do different measures of biological aging measure the same thing? and (2) Do different measures of biological aging capture different information about healthspan? Findings suggest promise and challenges in research to develop surrogate endpoints for trials of geroprotective interventions.

**Andreas Simm** (Martin Luther University Halle-Wittenberg) presented “Biomarkers of Ageing: the what-where from-when questions”.

**Tamas Fülöp** (University of Sherbrooke) presented “Are there any reliable biomarkers for immuno-senescence?”. Aging is accompanied by many physiological changes including those related to the changes in the immune system. These changes are called Immunosenescence which is accompanied by the Inflamm-aging phenomenon. Many biomarkers have been proposed to describe these age-associated changes in the immune system. One of the most consistent is the chronic Cytomegalovirus infection. Most of the elderly are affected in developed countries which about 70% and in developing countries about 100% at the age of 80. Despite the numerous studies there is no consensus which role the recurrent CMV infections play in the alterations of the immune system namely in the Inflamm-aging process and in the more consistent phenotypic alterations of T cells. Many experimental evidence supports that changes observed with aging (increase of memory CD8+ T cells, increase in pro-inflammatory cytokine mediators) may be related to chronic CMV infections. However, this infection was also showed to be a promoter of a better immune response in some cases e.g. vaccination. There is still debate whether CMV infection causes the immuno-senescence which in this case would suggest that aging of the immune system is nothing else, but just a viral infection. In contrast, if this is just a concomitant phenomenon thus in this case we should target other biomarkers which would represent the immuno-senescence. There is much confusion between diseases and biological aging, thus one of the main tasks is to define biological aging. Longitudinal studies combined with healthy very old elderly persons studies may give some clue to this and may help to find earlier biomarkers to reflect biological aging changes and eventually modify them.

**Joao Pedro de Magalhaes** (University of Liverpool) presented on “Gene expression profiling for the discovery of biomarkers of ageing”. There is widespread interest in identifying biomarkers of ageing in order to accelerate basic and translational research. Our lab has employed various gene expression profiling approaches to identify molecular signatures that can be used as biomarkers as well provide functional insights on ageing and its manipulation. We performed a meta-analysis of ageing gene expression profiles using microarray datasets from multiple mammalian tissues, which revealed several conserved molecular signatures of ageing. We also applied our network and meta-analysis methods to dietary manipulations of ageing, in particular caloric restriction (CR), and identified candidate genes and processes strongly associated with CR in mammals. Moreover, we have been employing whole transcriptome profiling (RNA-seq) to study ageing and its manipulation by diet, which has significant advantages when compared to microarrays. Lastly, to catalogue and help understand ageing changes, we developed the Digital Ageing Atlas (http://ageing-map.org/), a one-stop collection of human age-related data covering different biological levels (molecular, cellular, physiological, psychological and pathological).

**Arnold Mitnitski** (Dalhousie University) reported “Indices of biological aging as indicators of heterogeneity of people's health”. There is an increasing interest to assessing biological aging - the aging rates are different in individuals of the same chronological age. Some found attractive an idea that these differences reflect differences in “biological age”. How to estimate biological age is a matter of ongoing debates. The techniques differ also depending on which biomarkers are used to calculate of biological age. We discuss the different approaches to estimation of biological age based on biomarkers of aging of different nature: from the sub-cellular level to the level to the whole organism. We consider this issue from the heterogeneity of people at the same chronological age perspective, from which the index of biological age is only one of possible measures of such heterogeneity. We illustrate some challenges in assessing biological age, by comparing a few measures of heterogeneity: the indices of biological age, defined with and without chronological age, and the frailty indices. By providing head-to-head comparison of these measures we demonstrated that inclusion of chronological age in the measure of biological age is unnecessary, and also (at least in the data sample we present) inferior to the frailty indices. The latter are based on the higher number of biological and clinical data therefore more likely capture the essential characteristics of health and its changes during aging, than a rather restricted set of biomarkers used to calculate biological age score. Also, the items used to calculate the frailty indices are readily available (either from the routine blood tests or/and from clinical assessments), the algorithm of its assessment is simple, in contrast to the obscured statistical techniques of calculating the biological age scores from a relatively restricted number of continuous biomarkers. Extensive comparisons across different databases are required. We discuss the problem of integration of various biological/clinical in a unified index of biological age.

**Alexander Kulminski** (Duke University) reported “Can age-specific genetic effects be relevant to biological age?”. Living organisms are getting older and eventually die at a certain age. The actual time an organism has been alive refers to chronological age (CA). However, not all organisms die at the same chronological age even if they are of the same species. The idea of biological age (BA) is that the differences in lifespan of these organisms can be due to an internal clock. For humans, BA refers to how old that human seems. A problem, however, is how to quantify BA. A promising approach could be to express BA in terms of measurable phenotypes such as biomarkers. As phenotypes, biomarkers represent endpoints of a cooperative work of genes in an organism. Accordingly, BA could readily have a genetic origin. Does it necessarily imply that there should be specific genes regulating BA? The answer is not straightforward. Associations of BA with telomere length seems to support existence of BA-specific genes. However, studies of laboratory animals show that even genetically the same organisms can have dramatically different lifespan even if they are in the same, perfectly controlled environment. These and other findings suggest that BA can be modulated not only by the BA-specific genes but also by changes in the effects of other genes over CA. Studies show that epigenetic modifications can modulate the effects of genes over CA. What about genes themselves, do they influence the same phenotypes differently at different ages? It is often assumed that genes have age-independent effects on phenotypes. In this presentation, we will discuss evidence of the changing role of genes in human phenotypes with age.

**Alex Zhavoronkov, Evgeny Putin, Alex Aliper, Mikhail Korzinkin** (Pharmaceutical Artificial Intelligence Department, Insilico Medicine, Inc.) presented a talk on “Gamification of data collection for deep learning and biomarker development”. Since the publication of Horvath's epigenetic aging clock in 2013, the field of aging biomarkers is rapidly evolving with epigenetic and transcriptomic markers developed to accurately predict chronological age of the patient. However, since these tests are reasonably new and expensive, the number of samples with clinical outcomes is still low making it difficult to assess the biological relevance and facilitate for rapid clinical propagation.

One data type, medical professionals have a lot of experience with, which is abundant, cost-effective and actionable is clinical blood and urine tests commonly including basic blood biochemistry and cell counts. Millions of clinical profiles with blood biochemistry data are available worldwide providing sufficient number of samples to train deep neural networks.

We obtained a large data set of standard blood tests with 41 parameters linked to age and sex of the patients from Invitro Laboratories, one of the largest providers of laboratory services in Eastern Europe. The data set contained over 60,000 samples coming from routine checkups and excluded sources of patients with diseases like hospitals and medical institutions. We trained 21 single class deep neural networks ensembled using a stacking model to predict the patients' chronological age with 83.5% epsilon-accuracy r of 0.91 with R(2) of 0.82 and mean absolute error (MAE) of 5.55 years.

Following on the footsteps of Microsoft's http://www.how-old.net, we built a website http://www.aging.ai inviting people from all over the world to submit their test results in order to guess their age. From January 15 to March 31st over 1,500 people participated in the study. In addition to blood tests we asked to provide height, weight and smoking status, the new parameters that are being evaluated for the future studies. We plan to expand Aging.AI system to work with urine, transcriptomic and imaging data and welcome collaborations in building comprehensive and actionable biomarkers of aging.

**Ivan V. Ozerov** (Senolytics Department, Insilico Medicine, Inc.) presented “iPANDA identifies the pathway signature in senescent cells: a step forward to development of novel senolytic drugs”. Cellular senescence combined with an inability of immune system to effectively eliminate senescent cells leads to persistent accumulation of senescent cells in an aging organism. On the other hand, senescent cells represent a constant danger to the cell population as far as they partly lose their functions and induce malfunctioning of surrounding cells. As senescent cells accumulate in even greater numbers over the years, the whole tissues gradually loose their specific properties. Such process results in developing of aging phenotype and encourages the risk of malignant transformation in the affected cells. Hence therapies aimed to selectively eliminate senescent cells have a potential to slow down age-related changes in tissues and body in whole as well as to reduce the risks of cancer generation. Recently three low-molecular compounds which demonstrate an ability to selectively eliminate senescent cells in various tissues were proposed. This novel class of prospective drugs is referred to as senolytics.

In this study, we apply our recently-developed approach to large-scale transcriptomic data analysis in silico Pathway Activation Network Decomposition Analysis (iPANDA) to identify pathway signatures of senescent cells in various tissues and pathway signatures of known senolytic drugs. iPANDA algorithm is specifically designed to obtain robust results when analyzing transcriptomic data from multiple sources. Thus, we were able to extract the common tissue-independent features of senescent cells including downregulated anti-apoptosis signalling networks as well as several tissue-specific features of cellular senescence. In order to find novel compounds with senolytic properties we utilized this information for obtaining a list of prospective protein targets. A list of about 100 low-molecular senolytic candidates was derived partly from the pharmacophore-based scanning of proposed protein targets and partly from a list of known drugs which selectively affect the identified pathways involved in cellular senescence. At present moment, the validation process of these compounds in cell culture experiments is on the way.

**Ksenia Lezhnina** and colleagues (Insilico Medicine, Inc.) presented “Signaling pathways signature of sarcopenia identified by iPANDA algorithm.” Sarcopenia is a losing muscle mass and function with aging. Decreased strength and power of muscle function may contribute to higher risks of accidents among older people and affects quality of life. Until recently sarcopenia was not even considered as a pathological condition and as a consequence clinical definition and diagnostic criteria is poorly developed. Mechanism underlying sarcopenia is extensively investigated but still not fully understood. In order to study this we compare transcriptomic profiles of muscle tissues from young and old people, both women and men. We assume that aging process starts from the fourth decade of life. We apply a new algorithm in silico Pathway Activation Network Decomposition Analysis (iPANDA) to transcriptomic data to find signaling pathway signatures of aging in muscle tissues. Common pathway signatures can be considered as a target for development of new approaches for sarcopenia treatment.

**Artem Artemov** and colleagues (Insilico Medicine, Inc.) presented “*In silico* screen for drugs increasing cancer immunotherapy success rates”. Cancer immunotherapy has been shown to be extremely efficient. Unlike traditional targeted therapy, it can lead to a complete remission rather than a few months increase in lifespan. Unfortunately, it is beneficial for only a small subset of patients. In this work, we performed *in silico* screening of compounds which can be administered in combination with anti-PD1 immunotherapy to increase immunotherapy success rate. We collected publicly available transcriptome data for tumours responding and not responding to immunotherapy. Next, we applied iPANDA, Insilico medicine pathway analysis algorithm, and deep-learning based approach to identify a transcriptomic signature predicting the success of immunotherapy in a particular tumour. Finally, we analyzed drug-induced transcriptome effects to screen for the drugs which could robustly drive transcriptomes of tumour cells from non-responsive state to the state responsive to immunotherapy. Among the top-scoring drugs we found known compounds used in combination with cancer immunotherapy. Interestingly, we also found a compound which was a close chemical analog to a compound used in cancer immunotherapy, but hadn't itself been studied for this indication. This approach, after preclinical and clinical validation, may lead to improved cancer care and dramatic lifespan increase of cancer patients.

**Polina Mamoshina** and colleagues (Insilico Medicine, Inc.) presented “*In silico* modeling of human skin permeability of compounds with possible geroprotective activity” Despite the fact that many chemicals demonstrate potential skin rejuvenating activity, skin aging is still an incurable process. The major problem is that most of the anti-aging compounds have low skin penetration level and so bioavailability. Dermal absorption rate could be estimated by *in vitro* and *in silico* approaches. We utilized a set of Machine Learning techniques to predict skin permeability coefficient (Kp) of compounds with skin geroprotective activity. K-nearest neighbors algorithm showed the best performance with *r=*0,94, *R^2^=*0,86, *MAE*=0,29. This new approach may lead to a development of new effective remedies that could slow down or even reverse skin aging.

**Jane Schastnaya** (Insilico Medicine, Inc.) presented “iPANDA for biomarker identification in aging-associated hair loss”. In silico Pathway Activation Network Decomposition Analysis (iPANDA) is a novel biomathematical method, which has a potential to be a universal tool for pathway activation analysis in the treatment of anti-aging diseases and for the identification of actionable targets for therapy. It can be used for the analysis of any physiological, stress, malignancy and other perturbed conditions at the molecular level. We showed the results of a qualitative analysis of signaling pathway activation state in androgenetic alopecia. Some of these signaling pathways may serve as targets for the treatment of hair growth disorders. For example, recent findings demonstrated that inhibition of JAK-STAT signaling pathway can promote hair growth. Further progress in research will lead to the increasing insight into pathways involved in pathological hair cycling.

**Alexander Fedintsev** and **Alexey Moskalev** (Moscow Institute of Physics and Technology) presented “Markers of cardiovascular health for human chronological and biological age estimation”. The aging process is unavoidably associated with a decline in functional physical capacity. A lot of anti-aging interventions are known but they were tested only on model organisms so it is very important to develop simple, cheap and accurate method of biological age estimation which can be used to control anti-aging therapy efficacy. To develop such a method, we performed analysis using machine learning approach to reveal parameters which are mostly associated with chronological age and selected four of them. These four predictors which showed highest importance are: complex intima media thickness, arterial stenosis, augmentation index, and pulse wave velocity. The combined index composed of these four parameters explained up to 61% of variance in age, more than other biomarkers of age like telomere length or glycans. We also measured median absolute error which was 5.12 years for women and 5.46 years for men. These four parameters reflect aging of cardiovascular system. Cardiovascular diseases are the major cause of age-related mortality so we assume that our method is good estimation not only of chronological but also of biological age. In addition, this method uses medical equipment which is widely represented in modern clinics.

**Peter Fedichev** (Gero, Inc.) in his report “Target and biomarker identification platform to design new drugs against aging and age-related diseases” studied fundamental aspects of aging to develop a mathematical model of gene regulatory network. We show that aging manifests itself as an inherent instability of gene network leading to exponential accumulation of regulatory errors with age. To validate our approach, we studied age-dependent omic data such as transcriptomes, metabolomes etc. of different model organisms and humans. He builds a computational platform based on our model to identify the targets and biomarkers of aging to design new drugs against aging and age-related diseases. As biomarkers of aging we choose the rate of aging and the biological age since they completely determine the state of the organism. Since rate of aging rapidly changes in response to an external stress, this kind of biomarker can be useful as a tool for quantitative efficacy assessment of drugs, their combinations, dose optimization, chronic toxicity estimate, personalized therapies selection, clinical endpoints achievement (within clinical research), and death risk assessments. According to the model were proposed a method for targets identification for further interventions against aging and age-related diseases.

**Anatoliy Yashin** (Duke University) reported “Lack of Replication in GWAS of Complex Traits: Insights for Efficient Analyses of Human Aging and Longevity”. The genome-wide association studies (GWAS) performed during the last decade detected a large number of SNP loci that influence variability of human aging, health, and longevity related traits. While many of these findings were confirmed in analyses of independent populations many other identified associations remain non-replicated. The persistent non-replication of the research results indicates a problem that needs immediate attention. The purpose of this paper is to investigate the possible contribution of population genetic structure to this problem. We reviewed the results of recent GWAS of longevity related traits and investigated forces and mechanisms that form genetic structures of human populations. We found that these structures may differ in populations used in genetic association studies. We investigated the role of linkage disequilibrium (LD) between functional SNPs on estimates of the effects of these SNPs on mortality risks. We found that the estimates of genetic associations of minor allele in the first locus with lifespan as well as age trajectories of mortality rates for carriers and non-carriers of this allele are strongly modulated by the levels of LD between the two loci. Depending on the LD levels between these SNPs the same genetic variant may have positive, negative, or no association with longevity related trait. The estimated associations may also change with increasing age from positive to negative and vice versa. The results of these analyses indicate that the difference in LD between the two functional SNP loci in the two study populations may contribute to the lack of replication of the results of genetic association studies of human longevity related traits. These results also show that studying LD patterns around functional SNPs, as well as effects of haplotypes on longevity related traits may contribute to better understanding the genetic nature of these traits.

**Alexander Maslov (**Albert Einstein College of Medicine) presented “Quantitative assessment of genome integrity by high-throughput sequencing: application for aging research”. Aging is a complex trait governed by both genetic and environmental factors. While environmental exposure promotes genome instability, a major driver of the aging process, genetically determined mechanisms of genome maintenance act in the opposite direction and promote longevity. The idea that environmental hazards promote aging is not novel. Twin studies have demonstrated that environmental factors play an important role in the development of degenerative diseases associated with aging and that the relative contribution of non-genetic factors increases in advanced ages. However, until recently these studies had a mostly descriptive character; now since the advent of new technologies in genetic research, predominantly next-generation sequencing, we are capable of taking this a step further and directly assess genome integrity in primary cells or tissues of any species.

**Matt Kaeberlein** (University of Washington) presented “Effects of transient mid-life rapamycin treatment on lifespan and healthspan”. The FDA approved drug rapamycin increases lifespan and improves measures of healthspan in rodents [1]. Nevertheless, important questions exist regarding the translational potential of rapamycin and other mTOR inhibitors for human aging, and the optimal dose, duration, and mechanisms of action remain to be determined [2]. Here I will report on studies examining the effects of short-term treatment with rapamycin in middle-aged mice and dogs. We find that transient treatment with rapamycin is sufficient to increase life expectancy by more than 50% and improve measures of healthspan in middle-aged mice. This transient treatment is also associated with a remodelling of the gut microbiome, including dramatically increased prevalence of segmented filamentous bacteria in the small intestine, along with a dramatic shift in the cancer spectrum in female mice. In dogs, we have defined a dose of rapamycin that is well tolerated, and initial results are consistent with improvements in age-associated cardiac function similar to those observed in rapamycin-treated mice. These data suggest that a transient treatment with rapamycin may yield robust health benefits in mice, dogs, and perhaps humans.

**Stephen Spindler** (University of California, Riverside) presented “Flies, mice and humans, and the search for longevity therapeutics”.

**James Mitchell** (Harvard T.H. Chan School of Public Health) reported “Increased endogenous hydrogen sulfide as a conserved mechanism of longevity extension”.

**Maxim Skulachev** (Moscow State University) presented “Development of mitochondrially-targeted geroprotectors: from the molecular design to clinical trials and marketing strategy”. Research and development of geroprotectors is always challenging when the project passes from theoretical and laboratory work to routine drug development (preclinical and clinical trials and medical authority approvals). In this talk, we present an example of an anti-ageing RnD project aimed on creation of geroprotector drugs on the basis of rechargeable mitochonrially-targeted antioxidants. Our strategy is to get the potential gero-protector approved as a drug against a certain age-related disease, and then to expand the list of indications for this pharmaceutical to other traits of ageing. We synthesized a series of novel organic compounds, namely, cationic, membrane-permeable derivatives of plastoquinone. Preclinical studies gave very promising results in several animal models of age-related diseases, including eye-diseases, neurodegeration and inflam-mation. Our first pharmaceutical was designed for local administration (in the form of eye-drops) to speed up the process of clinical development and to get the clinical data faster. At the current stage of the project our first drug Visomitin (Rx eye drops with antioxidant SkQ1 helping in such age-related diseases as dry eye syndrome and cataract) has been approved and marketed in Russia and successfully passed phase II clinical trials in US. Systemic oral form of SkQ1 has entered clinical trials in Russia and completed preclinical program in US and Canada. We consider our project to be a valuable attempt to slow down human aging by a mitochondrial approach.

**Sibylle Jager** (L'Oreal) presented “Longevity compounds for skin anti-aging: promising strategy or false hope”.

**Andrey Voronkov** (Moscow Institute of Physics and Technology) presented “Polypharmacological geroprotectors for healthy longevity”. In modern drug discovery, the concept of polypharmacology has attracted substantial attention. In this concept one multi-targeting drug, or drugs combination is used for targeting of several, complementary biotargets, which can result in synergistic effect. Synergism results from increase of the efficiency due to the complex interactions between biotargets and networks, regulated by them. Synergistic effects can assist the reduction of the therapeutic dose and correspondingly reduce the side effects. Therefore multi-targeting represents a very prospective direction for the drug discovery, which can be used for the treatment of the complex and difficult for the treatment diseases, while the side effects of such therapies will be reduced in comparison to single-targeting drugs combinations. One example of such diseases are oncologic diseases without cures. Another example of such condition is aging. We are working on the universal methods and approaches for design of the small molecules, capable to regulate complex biological processes, such as aging and cancer, while at the same time such therapies will expose organism to minimum of the possible side effects.

**Alexey Moskalev** (Institute of Biology of Komi Science Center of Russian Academy of Sciences) presented “Geroprotectors study on Drosophila model”. To date, more than 200 substances that prolong the life of model organisms have been reported in the literature (geroprotectors.org). Reducing the cost and improving the efficiency with which increasingly large amounts of data from model organisms can be applied to humans will be critical to progress in the development of human geroprotectors. For this purpose, we have to come to an agreement what should be considered applicable to human geroprotectors. Primary selection criteria for potential geroprotector:

1. Increased lifespan in models or human. The increase in lifespan is not always accompanied by positive changes in the quality of life, and additional criteria for geroprotectors is needed, and discussed below
2. Amelioration of human aging biomarkers (http://ageing-map.org)
3. Acceptable toxicity
4. Minimal side effects
5. Improving health-related quality of life
6. Secondary selection criteria for potential geroprotector:
7. Evolutionary conservatism of target or mechanism of action (agingchart.org)
8. Reproducibility of geroprotective effects on different model organisms
9. Simultaneous influence on several aging-associated causes of death in mammals
10. Increase of stress resistance

**Vladimir Anisimov** (N.N. Petrov Research Institute of Oncology) presented “Light desynchronosis, cancer and aging”. Light-at-night has become an increasing and essential part of modern lifestyle and leads to a number of health problems, including excess of body mass index, cardiovascular diseases, diabetes and cancer. Exposure to constant illumination was followed by accelerate aging and tumorigenesis in female CBA, 129/Sv and transgenic HER-2/neu mice. Male and female rats were subdivided into 4 groups and kept at various light/dark regimens: standard 12:12 light/dark (LD); natural lighting of the North-West of Russia (NL); constant light (LL), and constant darkness (DD) since the age of 25 days until natural death. Exposure to NL and LL accelerated estrous function switch-off, induced metabolic syndrome and tumors, reduced life span rats as compared to the standard LD regimen. Melatonin given in nocturnal drinking water prevented adverse effect of LL and NL. The LL regimen accelerated colon carcinogenesis induced by 1,2-dimethylhydrazine (DMH) in rats whereas DD or the treatment with melatonin alleviated the effect of LL. The LL regimen accelerated whereas the DD regimen inhibits both mammary carcinogenesis induced by N-nitrosomethylurea in rats. Nocturnal drinking of melatonin increased the mean life span in female CBA, SHR, SAMP-1 and transgenic HER-2/neu mice. Melatonin inhibited spontaneous or chemically induced carcinogenesis in mammary gland, colon, uterine cervix and vagina, lung, skin and soft tissues. Gene expression profile study in the heart and brain of melatonin-treated CBA mice has shown that genes controlling the cell cycle, cell/organism defense, protein expression and transport are the primary effectors for melatonin. Melatonin has also increased the expression of mitochondrial genes, which correlate with its ability to inhibit free radical processes. Meta-analysis of clinical data has shown positive effect of melatonin in treatment of cancer patients. Thus, we believe that melatonin may be used for prevention of premature aging and cancer development.

**Mikhail Shaposhnikov**, **Alexey Moskalev** (Institute of Biology at Komi Science Center of Russian Academy of Sciences) and **Peter Fedichev** (Gero Ltd., Hong Kong) presented “Effect of Sun/klaroid gene deletion on lifespan, stress resistance, locomotor activity and fertility in Drosophila melanogaster”. Accumulation of the inner nuclear envelope the Sun1 protein leads to severe tissue pathologies with decreased lifespan in mice with Hutchinson-Gilford progeria syndrome. Progeroid animals that are deficient for Sun1 show markedly reduced tissue pathologies and enhanced longevity. However, the role of SUN1 in normal aging is not clear. Here we studied the effect of Drosophila melanogaster klaroid/Sun gene deletion, on lifespan, stress resistance (to starvation, paraquat, hyperthermia, and ionizing radiation), age dynamics of locomotor activity and fecundity. It was found that hetero- and homozygous for the klaroid/Sun deletion Drosophila melanogaster males and females have increased median and maximum lifespan. It was noted that lifespan in flies homozygous for studied deletion was longer than in heterozygotes flies. The effect of the klaroid/Sun deletion on stress resistance depends on the genotype and the sex. In males, the klaroid/Sun deletion leads to an increase in locomotor activity, while in females there were no changes in locomotor activity. The increased female fecundity was detected in individuals with the mutation in the klaroid/Sun gene for both homo- and heterozygote. Thus, the increase in lifespan in klaroid/Sun Drosophila melanogaster mutants is not accompanied by the negative effects on fecundity, stress resistance and locomotor activity.

Accumulation of the inner nuclear envelope the Sun1 protein leads to severe tissue pathologies with decreased lifespan in mice. However, animals that are deficient for Sun1 show markedly reduced tissue pathologies and enhanced longevity. Here we studied the effect of Drosophila melanogaster klaroid/Sun gene deletion, on lifespan, stress resistance (to starvation, paraquat, hyperthermia, and ionizing radiation), age dynamics of locomotor activity and fecundity. It was found that hetero- and homozygous for the klaroid/Sun deletion Drosophila melanogaster males and females have increased median and maximum lifespan. It was noted that lifespan in flies homozygous for studied deletion was longer than in heterozygotes flies. The effect of the klaroid/Sun deletion on stress resistance depends on the genotype and the sex. In males, the klaroid/Sun deletion leads to an increase in locomotor activity, while in females there were no changes in locomotor activity. The increased female fecundity was detected in individuals with the mutation in the klaroid/Sun gene for both homo- and heterozygote. Thus, the increase in lifespan in klaroid/Sun Drosophila melanogaster mutants is not accompanied by the negative effects on fecundity, stress resistance and locomotor activity.

**Ekaterina Proshkina** with co-authors (Institute of Biology of Komi Science Center of Russian Academy of Sciences) presented “The influence of the activity of DNA damage recognition and repair genes on the lifespan of Drosophila melanogaster”. DNA damage is commonly occurs in the course of normal metabolic processes and accompanied aging. At the same time, a number of facts evidenced the relationship between DNA repair efficiency and longevity of organisms. In the present research authors focused on the determination of lifespan by activity of different DNA damage recognition and repair genes using the fruit fly D. melanogaster as a model object. In the most cases, mutations in genes, which encode proteins providing DNA damage recognition (ATM and ATR homologues), regulation of stress response (p53 and Gadd45), DNA excision repair (XPC, XPF, PCNA homologues), and double-strand break repair (BLM, Rad50, Rad54, XRCC3 homologues), sufficiently decreased D. melanogaster lifespan. Thus, dysfunction of DNA repair genes impairs organism vitality and reduces lifespan. An alternative approach is the screening for genes the overexpression of which increases the lifespan. Overactivation of DNA repair genes encoding enzymes that coordinate the recognition of DNA damage (HUS1, CHK2 homologues), base and nucleotide excision repair (AP-endonuclease-1, XPF and XPC homologues), double-strand break repair (BRCA2, XRCC3, KU80 and WRNexo homologues) led to both the positive and negative effects on D. melanogaster lifespan dependent on driver, stage of induction, gender and the role of a gene in the DNA repair process. Conditional ubiquitous and constitutive neuron-specific overexpression of investigated DNA repair genes negatively changed lifespan. Constitutive ubiquitous and conditional neuron-specific overexpression of genes involved in DNA damage recognition and repair mainly prolongs lifespan. Most beneficial effects were found out for homologous of genes HUS1, CHK2, XPC and XPF, that execute regulatory functions and provide the excision repair process. At the same time, negative effects of the stimulation of DNA repair genes can be associated with the absence of appropriate epigenetic regulation and the excessive energy expenditure.

**Ekaterina Lashmanova** and colleagues (Moscow Institute of Physics and Technology) presented “The effects of novel 2-selenohydantoin derivatives on lifespan and stress resistance of nematodes”. Selenium is an essential element for human health. It plays an important role in thyroid hormone metabolism and immune system. It also possesses antitumor effects. Furthermore, selenium is present in the enzyme glutathione peroxidase, which detoxifies peroxides and hydroperoxides, and selenium availability regulates enzyme's activity. Recently, 12 novel 2-selenohydantoin derivatives were synthetized and their antioxidant activity was revealed using electrochemical methods (Ivanenkov et al., 2016). The goal of the present study was to investigate the effects of these compounds on lifespan and stress resistance of nematodes.

The experiments were performed using the wild type N2 Caenorhabditis elegans. Nematodes were cultured in liquid medium at 20°C in 96-well plates. Selenium-containing compounds were added in final concentrations 100, 10 and 1 μM on the first day of adulthood. Each experiment was performed five times. In addition, the effects of these compounds on the survival of nematodes under stress conditions was studied.

The addition of 2-selenohydantoin derivatives didn't significantly effect resistance of nematodes to oxidative stress (100 mM paraquat) and heat shock (33°C). Eleven 2-selenohydantoin derivatives out of twelfth did not have any significant effects on median and maximum lifespan of nematodes or effects were not stable. At the same time, one of the studied compounds in concentration 1 μM significantly increased the median lifespan of *C. elegans* by 11%. However, the effects of this compound in other concentrations were not detected.

These results indicate the prospects of search of geroprotectors among 2- selenohydantoin derivatives.

**Ilya Solovev**, **Eugenia Dobrovolskaya** with co-authors (Institute of Biology of Komi Science Center of Russian Academy of Sciences) presented “Effect of neuron-specific circadian clock gene overexpression on *Drosophila melanogaster* lifespan”. Genes of circadian rhythms change their expression during aging of different organisms. We analyzed available transcriptomic data from different on-line bases and compared circadian genes' expression profile changes in animals (*Caenorhabditis elegans*, *Drosophila melanogaster*, *Mus musculus, Homo sapiens, Heterocephalus glaber, Strongylocentrotus purpuratus* and *Balaena mysticetus)* showing various aging rates. We investigated from the datasets that expression profiles of circadian genes in heads of old *Drosophila melanogaster* are almost identical with the profiles in young imagos after ingestion of prooxidant, with the exception of *tim* and *pp2A-B'* genes. In addition, such genes as *clk, per, cry, pdp1, vrille, cwo* decreased their expression levels in normally aging individuals, when *cyc, tim, bdbt, sgg, pdf* showed an increase. These findings have led us to an idea of normalizing expression profiles of circadian oscillator elements to compensate potential aging-associated changes during all lifespan. Primarily, we overexpressed *clk, per, cry, cyc* and *tim* using neuron-specific RU486-inducible system, this resulted in the increase of median life expectancy (10%) in *tim-* and *cry12*-overexpessing females. Median lifespan of female fruit flies overexpressing *per10* was 5.4% longer than in control group. Noteworthy, overexpression of *clk* shortened (−10%) only female's lifespan. 4% augmentation of median life expectancy was observed for males overexpressing *per24* and *cyc*.

**Eugenia Dobrovolskaya, Ilya Solovev** with co-authors (Institute of Biology of Komi Science Center of Russian Academy of Sciences) presented “Effects of caloric restriction on lifespan of *Drosophila melanogaster* individuals with tissue-specific overexpression of circadian clock genes”. The aging process is associated with changes in the expression level of various genes. Genes forming a system of “biological clock” of the body are not an exception, it evidenced by the worsening with age in physiological rhythms and aperiodizm of sleep and wakefulness cycles in old individuals. The molecular clock found in each cell of the peripheral tissues of multicellular organisms. A main environmental factor connected to the rhythms of biological processes is light, with daily and annual variations in the light intensity are associated with such phenomena as sleep, physical activity, rest, growth, reproduction, sexual behavior, moult and migration. Most human genes of circadian rhythms are evolutionarily conserved and have orthologs in the fruit fly *Drosophila melanogaster*. It was found that adults of *D. melanogaster* show the decrease in gene expression of photosensitive protein Cryptochrome with age, while its overexpression in old flies slows down the rate of aging. On the other hand, fruit flies with mutations in the genes of circadian rhythms are characterized by a reduced life span. The purpose of this study was to investigate wheather caloric restriction affected the life span of Drosophila melanogaster with overexpressed circadian rhythms' genes (Period, Timeless, Clock, Cycle, Cryptochrome). We chose UAS / GAL4 system to ensure conditioned (mifepristone-inducible) gene overexpression in flies' muscules, fat body and gut. Drosophila lines were placed on standard media with different caloric values and life span had been being observed once a day. The results of this study demonstrate the relationship of circadian rhythms' gene regulation mechanisms and caloric restriction response pathways.

**Darya Peregudova** with co-authors (Institute of Biology of Komi Science Center of Russian Academy of Sciences) presented “Chemical (formaldehyde, toluene, tcdd) and physical (ionizing radiation) factors influence on the physiological and genetic *Drosophila melanogaster* characteristics”. The changes of living organisms' physiological characteristics caused by various stressors are based on the cellular and molecular alterations. The reason for changes in the gene expression may be caused by the direct gene damage due to exposure, or activation of different mechanisms of an organism's biological structures damage recognition and stress response promotion. The actuality of the formaldehyde, toluene, TCDD and irradiation low doses effects studying is their widespread occurrence and negative impact on living organisms.

The aim of this work was to study changes in expression of stress response genes (Hsp70, Mus209 (PCNA), Mus210 (XPC), Rrp1, Brca2, spn-B, Ku80, PARP-1, Gadd45, Wrinkled / Hid, Sod1, Sod2, Catalase, MST-1, Cyp4e2), immune response genes (Drosomycin, Defensin, Metchnikowin) and genes associated with aging (dSir2, FOXO, JNK), caused by the exposure to low doses of dioxin (0.822 and 1.644 μmol/L), toluene (50 and 100 μmol /L), formaldehyde (7%, 14%), and irradiation (20 and 40 cGy), and to study the attendant changes in physiological characteristics (life span, locomotor activity, fertility) in Drosophila melanogaster male and female (*Canton-S* wild type strain). As a result of this work, it was showed that the above-mentioned impacts cause a significant increase in median life span of Drosophila melanogaster wild type strain *Canton-S* individuals (by 2-4%), there were no changes in female fertility, the locomotor activity in males was significantly increased. It is also have been shown that the biggest part of the studied genes increases its expression after exposure to low doses of formaldehyde, toluene, dioxin and irradiation.

**Nadezhda Zemskaya** (Institute of Biology of Komi Science Center of Russian Academy of Sciences) with co-authors presented “The relationship of lifespan and stress resistance of different *Drosophila* species”. The relationship of stress resistance and longevity was described based on the results of experiments with various lines of certain biological species. As a rule, long-lived lines are more resistant to various kinds of stress, and short lived mutants of various model organisms are hypersensitive to adverse environmental factors. Similar links can be determined by comparing different species with each other. However, this is a lack of most current research in this area, that comparisons are made between evolutionarily far-spaced species (rodents, bats, man, whales), and many identified patterns associated with aging may actually be only distantly related to the problem.

The aim of this work was to study the differences in stress resistance of several species of the *Drosophila* genus, which are significantly different in lifespans. We investigated 12 *Drosophila* species (*D. ananassae, D. austrosaltans, D. biarmipes, D. erecta, D. kikkawai, D. melanogaster, D. pseudoobscura, D. saltans, D. simulans, D. virilis, D. willistoni, D. yakuba*) provided by Dr. V. Gladyshev (Harvard Medical School, USA), and estimated their lifespan as well as resistance to oxidative stress (20 mM paraquat), hyperthermia (35°C) and starvation. Correlation analysis of stress resistance and longevity data was performed. Studied *Drosophila* species demonstrated different reaction to stressors. The extremely long lifespan and enhanced resistance to all investigated stressors was found for *D. virilis*. Additionally, relatively high survival was shown for *D. melanogaster* under oxidative stress, for *D. kikkawai* and *D. pseudoobscura* under hyperthermia, for *D. biarmipes* in the starvation condition. Obtained data revealed relationship between maximum lifespan and stress-resistance of *Drosophila* species.

**Evgeny Rogaev** (Vavilov Institute of General Genetics RAS) “Aging, Genomics, Alzheimer' Disease”.

**Sergey Kozin** (Engelhardt Institute of Molecular Biology, Russian Academy of Sciences) presented “Aged isoform of β-amyloid as biomarker and drug target of Alzheimer's disease”. Alzheimer's disease (AD) is closely associated with ageing. In view of the amyloid hypothesis, the key molecular event of AD is a structural transition of β-amyloid (Aβ) from the physiologically normal monomer state to soluble neurotoxic oligomers accumulating in the form of insoluble extracellular aggregates (amyloid plaques) in brain tissues. Zinc ions as well as ‘aged’ Aβ species present in the plaques are known to play a crucial role in triggering pathological conversion of endogenous Aβ. Isomerization of Asp7 is the most abundant age-related spontaneous non-enzymatic modification of Aβ. Our in silico, in vitro and in vivo studies have shown that Aβ species with isomerized Asp7 (isoAsp7-Aβ) significantly differ in their properties from healthy (non-modified) Aβ molecules. We have found that isoAsp7-Aβ might constitute a nucleation seed and initiate formation of the neurotoxic zinc-dependent Aβ oligomers, thus inducing development of cerebral amyloidosis and other pathological processes characteristic of AD. Moreover, the role of the Aβ metal-binding domain (the N-terminal region 1-16) as the minimal necessary and sufficient pathogenic unit of isoAsp7-Aβ has been strongly suggested. These findings allow to link the emergence of isoAsp7-Aβ due to Aβ ageing with the onset of age-related pathology, and to use isoAsp7-Aβ species as potential biomarkers and drug targets of early diagnosis and therapy of AD.

**Anatoliy Yashin** (Duke University) presented “Genetics of Alzheimer's Disease: Insights for Studying Connection among Health, Aging, and Longevity”. A number of independent genome-wide association studies (GWAS) of Alzheimer's disease (AD) detected highly significant associations of genetic variants from APOE and TOMM40 genes with this disorder. Epidemiologic studies detected connections of AD with other diseases such as cancer, Parkinson disease, type II diabetes, CVD, and others. A number of studies link AD with presence of viral and bacterial infections (e.g., herpes simplex type 1 virus). Other studies find association of AD with processes developing in aging brain. The existence of such connections indicates that GWAS of AD may also detect genetic variants associated with other diseases, and help investigate common genetic mechanisms that link AD and related pathologies. This may contribute to better understanding of the origin of AD and improve efficiency of corresponding preventive and treatment strategies. We performed GWAS of Alzheimer's disease (AD) using LOADFS data available from dbGaP. The analyses showed strong (genome-wide significant) genetic signals for SNPs from several genes on chromosome 19. These include CTB-129P6.4, CTB-129P6.7, PVRL2, TOMM40, APOE, APOC1 and BCAM. Associations of SNPs from HLA-DQB2, LINCO1006, DKFZp779M0652, RNASE11, RP11-14J7.6, MIR7154 were close to genome-wide significance. These results are obtained in joint and separate analyses of male and female data. SNPs detected in these analyses were also found in studies of human longevity (e.g., rs2075650). This SNP is downstream gene variant of the PVRL2 characterized as “poliovirus receptor-related 2 (herpes virus entry mediator B)”. This SNP is also an intron variant of TOMM40 gene. We reviewed evidence from other studies of AD and its connection with other health disorders and compared them with our findings. The results of these analyses indicate that development of AD involves several genetic pathways that have connection to development of other chronic pathologies.

**Rosie Freer** (Cambridge University) presented “A protein homeostasis signature in healthy brains recapitulates tissue vulnerability to Alzheimer's disease”. In Alzheimer's disease, the aggregation of amyloid-beta and tau in plaques and tangles spreads progressively across brain tissues following a characteristic pattern. Over two decades after the characterization of the progressive development of this disease, the mechanisms that govern the selective vulnerability of these tissues remain under debate. Using transcriptional analysis of healthy brains, we identify an expression signature that predicts – well before the onset – the tissue-specific vulnerability to disease. We obtain this result by finding a quantitative correlation between the histopathological staging of the disease and the specific expression patterns of the proteins that co-aggregate in plaques and tangles, together with those of the protein homeostasis components that regulate amyloid-beta and tau. Since this expression signature is evident in healthy brains, our analysis provides an explanatory link between a tissue-specific environmental risk of protein aggregation and a corresponding vulnerability to Alzheimer's disease.

**Natalia Stefanova** (Institute of Cytology and Genetics of Siberian Branch of Russian Academy of Sciences) “Prefrontal cortex transcriptomic indices of sporadic Alzheimer's disease in human and in a rat model”. Alzheimer's disease (AD) is the most prevalent neurodegenerative disease. We showed that senescence-accelerated OXYS rats represent a promising model of sporadic form of AD with the typical signs of disease: degenerative alterations and death of neurons, synaptic and mitochondrial dysfunction, hyperphosphorylation of the tau protein, accumulation of amyloid β, and the formation of amyloid plaques. Here, we aimed to compare the gene expression profiles of the prefrontal cortex from OXYS rats and Wistar rats as controls to identify the molecular mechanisms and the factors underlying disease progression. The transcriptome analysis was conducted at three stages of the disease (pre-symptomatic, symptomatic and progressive stage) in OXYS rats, using RNA-Seq technique. We identified marked differences in the prefrontal cortex transcriptome between the two rat strains already at the pre-symptomatic disease stage (> 600 genes), with an increasing of gene expression in the symptomatic stage (> 900 genes) and at the progressive stage (> 2 000 genes) in OXYS rats compared with age-matched Wistar rats. Gene ontology analysis of the transcriptional profile from OXYS rats showed marked changes of specific pathways involved in AD molecular pathway, as well as in mitochondrial and synaptic functions, protein phosphorylation, Ca2+ homeostasis, hypoxia, immune processes, and apoptosis. Then, we compared the gene expression profiles of the prefrontal cortex from human AD and OXYS rats. We demonstrated that transcriptional profile changes in the cortex as in human AD as well in OXYS rats, primarily due to mitochondrial dysfunction, synaptic plasticity and Ca2-signaling pathway. This study highlights a set of key genes and molecular pathway indices of disease which may prove useful in identifying potential disease modifiers responsible for the heterogeneity of human sporadic form of AD and which may represent valid therapeutic targets for ameliorating the disease course in humans.

**Olga Kovalchuk** (University of Lethbridge) presented “Chemo brain and aging – is there a link?”. It is projected that by 2030 newly diagnosed cancer cases will reach 21.7 million worldwide. The development of new chemotherapeutic agents and regimens for cancer therapy has led to increasing rates of survival in cancer patients and, therefore, it is important to ensure that cancer survivors suffer minimal side effects and have good life quality. Despite of undisputed benefits, chemotherapy causes a wide array of side effects, including central nervous system (CNS) toxicity. Chemotherapy-induced CNS side effects impact the cognitive domains of attention, memory, processing speed, and executive function, causing a condition that has been termed chemo brain. While the molecular and cellular mechanisms of chemo brain are not well investigated, the frequency and timing of its occurrence and their persistence suggest that chemo brain may be epigenetic in nature. We analyzed the effects of two commonly used cytotoxic chemotherapy drugs—cyclophosphamide (CPP) and mitomycin C (MMC) - on transcriptomic and epigenetic changes in the murine prefrontal cortex (PFC) and hippocampal regions. The key findings of our study are: (i) chemotherapy altered the gene expression profiles in the brain; (ii) MMC treatment resulted in accumulation of 8-oxodG, decreased global DNA methylation, and increased DNA hydroxymethylation in the PFC tissues of female animals; and (iii) the majority of the changes induced by MMC in the brain tissues resembled those that occur during the aging processes. This is the first study that suggests a link between chemotherapy-induced chemo brain and brain aging, and provides an important roadmap for future analysis.

**Vasily Popov** (Voronezh State University) presented “Methylene blue rejuvenates behavior and induces brain mitochondria biogenesis in aging mice”. Age-related brain dysfunctions are believed to be associated with deregulation of mitochondria functions increasing risks to develop neurodegenerative diseases (ND). Recently, mitochondria –targeting drug methylene blue has been drawing considerable interest as a potential treatment for ND. Despite well studied effects of MB at the level of isolated mitochondria and cells in several ND models, its effects on the functioning of non-diseased brain remains unexplored. We have compared the effect of per oral MB treatment on the behavior, mitochondria reactive oxygen species generation, and gene expression in adult and aged mice. We found that 15 month old mice manifested a decrease in physical endurance, spontaneous locomotor activity, and exploration concomitant with an increase in anxiety-related behavior, as compared to 7 month old mice. A 60 day MB treatment slow down these changes in 15 month old mice. There was no significant body weight change, oxygen consumption rate and RQ index in either adult or aged MB-treated mice. MB significantly increased the rate of ROS production in isolated brain mitochondria. The expression of several genes relevant to mitochondria biogenesis, bioenergetics, and antioxidant defense (NRF1, MTCOX1, TFAM, SOD2) was greatly suppressed in aged mice; it was restored by MB treatment. We hypothesize that the effects of MB may be mediated by its ability to increase H2O2 production in brain mitochondria, which in turn activates Nrf2/ARE signaling pathway and mitochondria biogenesis.

**Khusru Asadullah** (Editor in Chief of Advances in Precision Medicine) presented “Trends in translation medicine: the value of external innovation”. Collaborations between academic institutions and the diagnostics/pharmaceutical industry are increasingly being initiated and executed. It's assumed that these relationships could help to improve research and development productivity in industry, as well as enable academic institutions to better exploit the translational potential of their research. Identification and validation of targets and biomarkers, key elements of successful drug discovery, are challenging. Unfortunately, according published data are frequently irreproducible. Thus, we must not rely on published data only. Extensive joined efforts of multiple partners seem crucial to foster progress. Different models are used and required for different stages of the drug discovery and development process as well as for different kinds of targets and biomarkers.

